# Real-Time Scintigraphic Assessment of Intravenous Radium-223 Administration for Quality Control

**DOI:** 10.1155/2015/324708

**Published:** 2015-02-18

**Authors:** Chadwick L. Wright, J. Paul Monk, Douglas A. Murrey, Nathan C. Hall

**Affiliations:** ^1^Department of Radiology, The Ohio State University Wexner Medical Center, 410 West Tenth Avenue, Columbus, OH 43210, USA; ^2^Division of Medical Oncology, Department of Internal Medicine, The Ohio State University Wexner Medical Center, 410 West Tenth Avenue, Columbus, OH 43210, USA

## Abstract

Radium-223 (^223^Ra) dichloride is an approved intravenous radiotherapy for patients with osseous metastases from castration-resistant prostate cancer (CRPC). In addition to the therapeutic alpha radiation, there is additional ^223^Ra radiation generated which produces photons that can be imaged with conventional gamma cameras. No studies have evaluated real-time and quality imaging during intravenous ^223^Ra administration to verify systemic circulation and exclude ^223^Ra extravasation at the injection site. A retrospective review was performed for fifteen ^223^Ra administrations for CRPC patients which were imaged using a large field of view portable gamma camera (LFOVPGC) for the purposes of quality control and patient safety. Dynamic imaging of the chest was performed before, during, and after the ^223^Ra administration to verify systemic circulation, per institutional clinical protocol. Before and after ^223^Ra administration, a static image was obtained of the intravenous access site. Dynamic imaging of the chest confirmed systemic administration early during the 1-minute injection period for all patients. There were no cases of focal ^223^Ra extravasation at the site of intravenous access. These results verify that systemic ^223^Ra administrations can be quantified with real-time imaging using an LFOVPGC. This simple approach can confirm and quantify systemic circulation of ^223^Ra early during injection and exclude focal extravasation for the purposes of quality control.

## 1. Introduction

Targeted radiotherapy is a technique for treating primary malignancies and metastatic disease with intravascular administration of therapeutic radioisotopes, peptides, antibodies, and microspheres. ^223^Ra dichloride is approved for intravenous radiotherapy for patients with osseous metastases from castration-resistant prostate cancer (CRPC). ^223^Ra is an alpha-particle emitting radioisotope that mimics calcium and forms complexes with hydroxyapatite at areas of increased bone turnover, such as osseous metastases. ^223^Ra has a half-life of 11.4 days. Osseous metastases from CRPC are amenable to targeted radiotherapy using ^223^Ra and have the advantage of maximizing local alpha radiation effects to osseous metastases while minimizing radiation toxicity to adjacent normal bone and soft tissues [1, 2]. All patients referred for ^223^Ra radiotherapy must initially be evaluated for verification of osseous metastases using intravenous Technetium-99m (^99m^Tc) MDP bone scintigraphy or intravenous sodium fluoride-18 (Na^18^F) PET/CT to insure eligibility for therapy [3, 4]. A complete course of ^223^Ra dichloride radiotherapy involves intravenous administration of ^223^Ra every 4 weeks for 6 cycles. Most of the radioactivity produced by ^223^Ra results from the production of therapeutic alpha particles which travel only a very short distance in bone but are sufficiently energetic for therapeutic benefit. There is additional radiation generated by ^223^Ra which produces photons (i.e., 81 and 84 keV) that have potential to be imaged using routine clinical nuclear medicine imaging [1, 3, 5]. To date, no studies have evaluated real-time and quality imaging during intravenous ^223^Ra administration to verify systemic circulation and exclude ^223^Ra extravasation at the injection site.

## 2. Materials and Methods

This retrospective study was approved by the Institutional Review Board at the Ohio State University Wexner Medical Center (OSUWMC). Between October 2013 and January 2014, 15 radiotherapy administrations of ^223^Ra dichloride for 8 CRPC patients were performed and imaged using an institutional PGC imaging protocol for the purposes of quality control and patient safety. All 8 patients had received at least one prior administration of ^223^Ra.

### 2.1. Imaging Protocol

The imaging protocol utilized an LFOVPGC (DIGIRAD Ergo, DIGIRAD Corporation, Poway, CA, USA) operating under the Xenon-133 setting (photopeak of 81 keV with a 10% window) with a 128 × 128 matrix for preinjection and postinjection ^223^Ra planar and dynamic imaging. Low energy all purpose (LEAP) collimation was used. All images were subsequently processed using a Philips EBW workstation.

### 2.2. Preinjection Imaging

**Figure 1:**
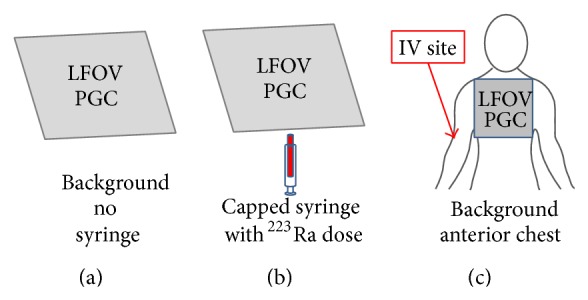
Preinjection LFOVPGC imaging (one minute per image) of the injection room background (left), capped syringe containing the ^223^Ra dose (middle), and anterior chest background (right). The LFOVPGC detector head is represented by either a gray trapezoid or gray square just overlying the imaging target.

Prior to injection, a 1-minute static image of the background activity in the injection room was obtained as well as 1-minute static images of the patient's capped syringe containing the treatment dose and the patient's anterior chest (Figure 1). The purpose was to confirm the presence of detectable ^223^Ra activity in syringe prior to injection and obtain background activity level assessments of the injection room and the patient's anterior chest before administration. The total preinjection LFOVPGC imaging time was 3 minutes (1 minute per image).

### 2.3. Dynamic Imaging during Injection

**Figure 2:**
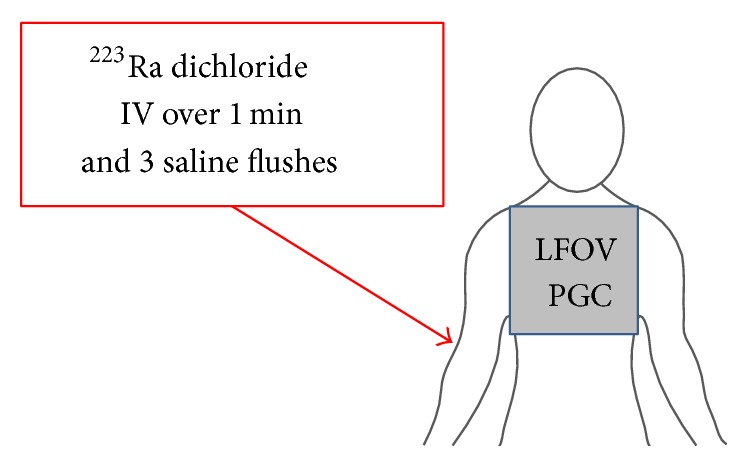
Dynamic LFOVPGC imaging of the anterior chest during intravenous administration of ^223^Ra and subsequent saline flushes (5-minute continuous imaging 6 sec/frame).

At the start of intravenous administration of ^223^Ra, dynamic imaging of the anterior chest was obtained at 6 seconds/frame for 5 minutes in order to image the angiographic phase during the entire injection and subsequent 3 saline flushes (Figure 2). The camera was allowed to acquire data for approximately 15–30 seconds before the IV administration began. The total dynamic LFOVPGC imaging time was 5 minutes for the dynamic imaging of the therapeutic IV administration.

### 2.4. Postinjection Imaging

**Figure 3:**
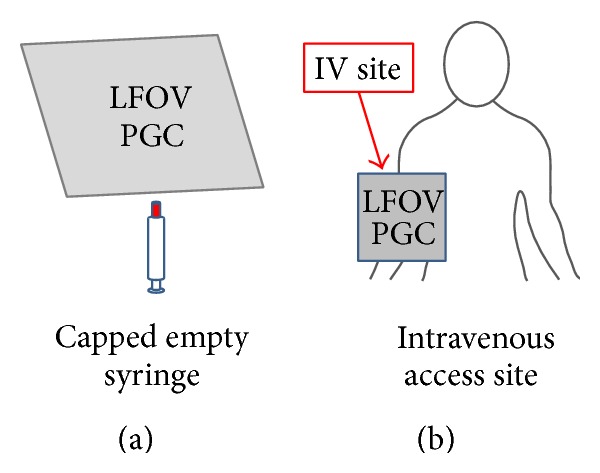
Postinjection LFOVPGC imaging of the capped empty syringe (left, 1 minute) and IV injection site (right, 1 minute).

Following slow IV injection of ^223^Ra and removal of the dedicated IV access, 1-minute static images, each of the patient's IV injection site and the capped empty syringe, were obtained (Figure 3). The purpose of these images was to confirm the presence or absence of any residual ^223^Ra activity in syringe or syringe cap after injection and to assess any gross extravasation of ^223^Ra at the IV injection site. The total postinjection LFOVPGC imaging time was 2 minutes (one minute per image).

### 2.5. Statistics

Unless otherwise indicated, all values are expressed as mean ± standard deviation. Linear regression analysis of LFOVPGC counts and syringe ^223^Ra activity was performed using JMP Pro 10.0.2 (SAS Institute, Inc.).

## 3. Results and Discussion

**Figure 4:**
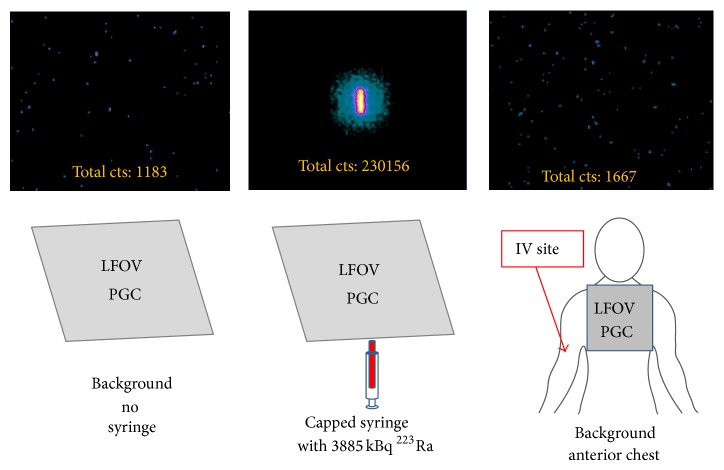
Preinjection LFOVPGC imaging of the injection room background (top left), capped syringe containing the ^223^Ra dose (top middle), and anterior chest background (top right). Total LFOVPGC counts for the entire field of view are provided for these 1-minute acquisitions (top row).

The average ± standard deviation for the ^223^Ra dichloride doses administered intravenously was 4662 ± 666 kBq (*n* = 15, range 3848–5994 kBq). During preinjection LFOVPGC imaging (Figure 4), the average injection room background activity was 1032 ± 61 counts (*n* = 15, range 916–1183 counts). The average background-corrected preinjection syringe activity was 291345 ± 49406 counts (*n* = 15, range 209633–389084 counts). There were two radiotherapy administrations (out of 15) in which the delivered ^223^Ra dichloride doses were provided in two syringes instead of one syringe (not shown and excluded from subsequent quantitative dynamic imaging analyses of the anterior chest). The average anterior chest background activity was 1722 ± 516 counts (*n* = 15, range 1016–2903 counts). The increased background counts for the anterior chest relative to the background counts for the injection room are consistent with the fact that all patients had received at least one prior ^223^Ra radiotherapy administration. This implies that ^223^Ra from the last radiotherapy administration had been incorporated into the osseous structures within the anterior chest field of view (presumably within osseous metastases) and some residual incorporated ^223^Ra was still detectable on the preinjection chest imaging. The average absolute difference in anterior chest and background activities was 691 ± 525 (*n* = 15, range 9–1878 counts).

**Figure 5:**
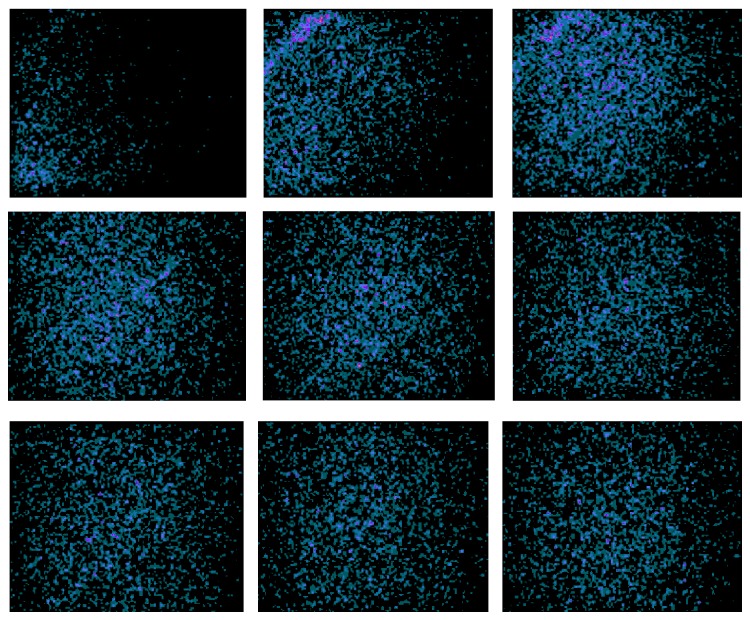
Dynamic images of the anterior chest during injection of 3848 kBq ^223^Ra via a right antecubital fossa IV site. Dynamic images were compressed into 30-second frames. First 30-second frame is in the upper left and progresses from left to right and top to bottom.

**Figure 6:**
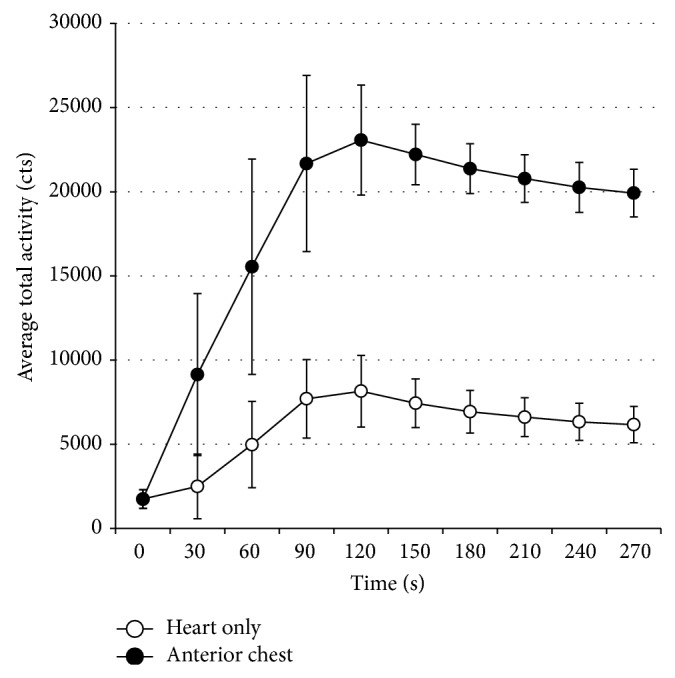
Average total activity counts for the heart and anterior chest ROIs for all 13 radiotherapy administrations using only single-syringe doses (*n* = 13). Quantitative ROI analysis confirmed systemic administration early during the 1-minute injection period and the time-to-peak ^223^Ra activity was at least 1 minute.

Dynamic LFOVPGC images of the anterior chest were compressed into 30-second frames (Figure 5). In all cases, dynamic imaging of the chest confirmed systemic administration early during the 1-minute injection period. Quantitative region-of-interest (ROI) analysis of the dynamic anterior chest imaging confirmed that the time-to-peak ^223^Ra activity was at least 1 minute for all radiotherapy administrations. ROIs were drawn around the heart and entire anterior chest field of view (including the heart) and total activity counts for each 30-second frame were measured. Dynamic ROI analysis confirmed that the time-to-peak ^223^Ra activity was at least 1 minute in all 13 single-syringe radiotherapy administrations (Figure 6).

**Figure 7:**
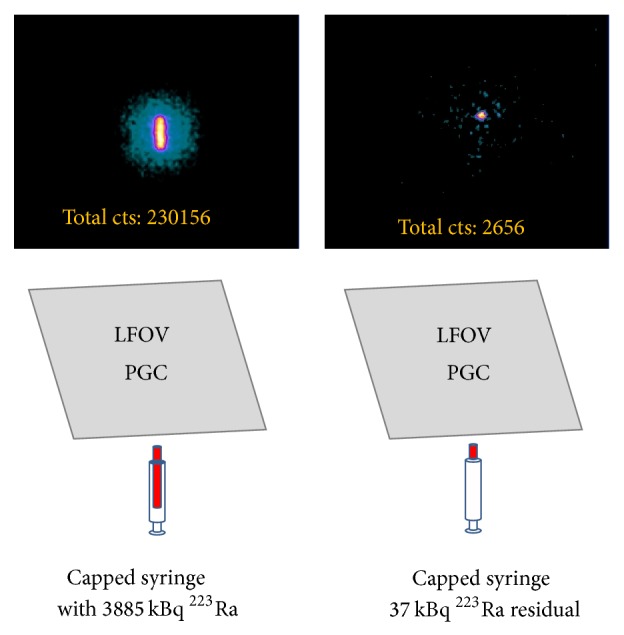
Preinjection LFOVPGC imaging of a capped syringe containing the ^223^Ra dose (top left) and subsequent postinjection imaging of the capped empty syringe (top right). There is a single discrete focus of activity in the capped empty syringe corresponding to 37 kBq of residual ^223^Ra activity within the syringe cap. Total LFOVPGC counts for the entire field of view are provided for these 1-minute acquisitions (top row).

During postinjection LFOVPGC imaging (Figure 7), the average ± standard deviation for the background-corrected postinjection syringe activity was 3312 ± 564 counts (*n* = 15, range 2656–4644 counts). The measured residual ^223^Ra activity was 37 kBq in all syringes and incidentally all residual activity localized to the syringe cap on imaging. Background-corrected preinjection and postinjection syringe activity positively correlated with the corresponding preinjection and postinjection syringe dose activity measurements (*r* ^2^ = 0.99, *n* = 30) (see the following equation):

(1) Background-corrected  syringe  activity (counts)  =87.66+62.21×Syringe  dose kBq.

**Figure 8:**
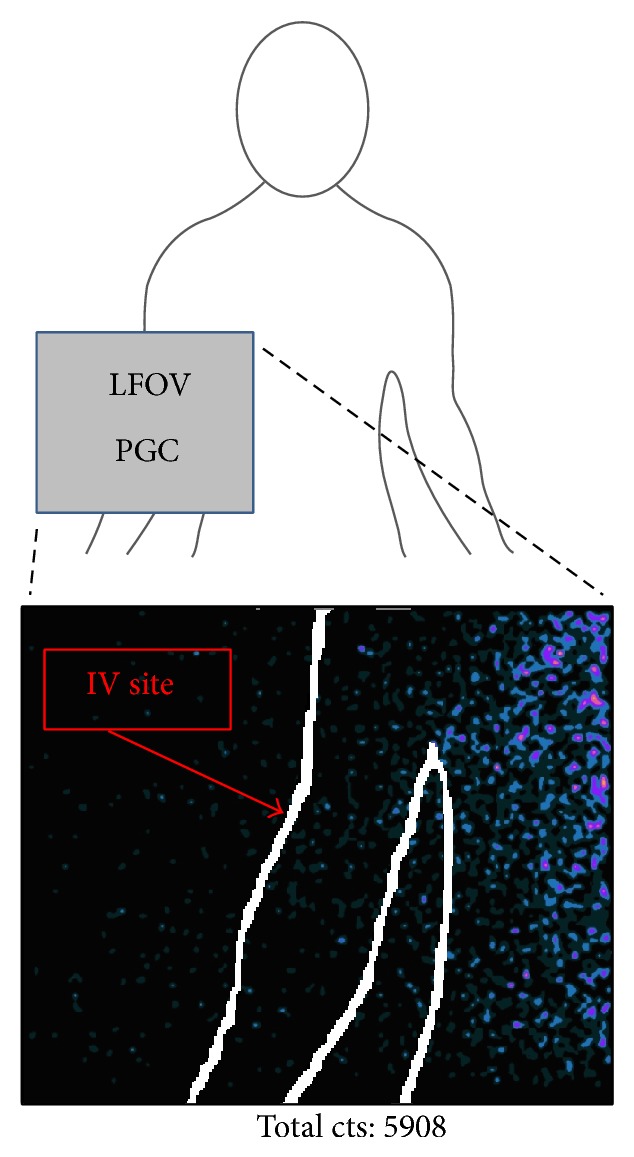
Postinjection LFOVPGC imaging of the patient's IV injection site (bottom, one-minute acquisition). A white outline of the patient's body contour has been manually superimposed on the bottom LFOVPGC image which demonstrates no evidence of focal ^223^Ra extravasation in the right antecubital fossa injection site but rather systemic ^223^Ra activity within the partially imaged chest and abdomen. Total LFOVPGC counts for the entire field of view are provided for this 1-minute acquisition (bottom).

There were no instances of focal ^223^Ra extravasation at the site of intravenous access (Figure 8). Given that 37 kBq of residual ^223^Ra activity within a syringe cap produced a discrete focus of activity on LFOVPGC imaging and that the vast majority of IV sites are superficially located (i.e., minimal soft tissue attenuation), it is likely that any focal extravasation of ≥37 kBq would be detectable at the IV site.

## 4. Conclusions

The demand for ^223^Ra dichloride is anticipated to increase significantly in the foreseeable future with potential expansion into women with osseous metastatic disease from breast cancer. Our results demonstrate that (1) systemic ^223^Ra administrations in CRPC patients can be dynamically imaged using a clinical LFOVPGC imaging system and (2) systemic ^223^Ra administrations can be further quantified with this real-time LFOVPGC imaging approach. Total time for this LFOVPGC imaging protocol is 10 minutes and the total patient imaging time is 7 minutes. This simple imaging approach can be used to quickly confirm and quantify systemic circulation of ^223^Ra during injection as well as evaluate focal soft tissue extravasation at the IV site for the purposes of patient safety and quality control.

Although no cases of focal soft tissue extravasation were identified in this study, our results indicate that injection site imaging could rapidly quantify focally extravasated ^223^Ra activity for subsequent monitoring and dosimetry. Serial imaging of the injection site (e.g., right antecubital fossa) and the contralateral noninjected limb (i.e., left antecubital fossa) would allow for quantitative assessment of ^223^Ra resorption and confirm when complete resorption was achieved (i.e., ^223^Ra activity in the injected limb approximates the activity in contralateral noninjected limb).

This real-time LFOVPGC assessment of the patient, dose, and injection site is a simple adjunct to routine survey meter evaluation by radiation safety or medical physics. There is sparse literature on the role of gamma camera imaging for quality assessment of intravenous radiotherapy administration. One study demonstrated the feasibility of gamma camera imaging to qualitatively assess Yttrium-90 bremsstrahlung activity using a phantom simulation of intravenous Yttrium-90-labeled antibody extravasation [6]. Thus, real-time LFOVPGC imaging can be easily adapted for real-time quality assessment of other radionuclide therapy administrations such as gamma-emitting intravenous radiotherapies (e.g., Samarium-153, Iodine-131 metaiodobenzylguanidine), bremsstrahlung-emitting intravenous radiotherapies (e.g., Strontium-90, Yttrium-90-labeled antibodies/peptides), and bremsstrahlung-emitting intra-arterial radioembolization therapies (e.g., Yttrium-90 containing resin or glass microspheres).

It remains to be determined in a prospective clinical trial throughout the course of ^223^Ra radiotherapy if there is any prognostic significance to serial quantitative assessment of the difference in measured residual activity between preinjection anterior chest and the background activity of the injection room (e.g., patients with significantly higher preinjection residual activity demonstrate improved clinical outcomes when compared with those patients with little or no preinjection residual activity).
